# Injection of Gelling Systems to a Layered Reservoir for Conformance Improvement

**DOI:** 10.3390/gels8100621

**Published:** 2022-09-29

**Authors:** Konstantin Fedorov, Alexander Shevelev, Alexander Gilmanov, Andrey Arzhylovskiy, Denis Anuriev, Ivan Vydysh, Nikita Morozovskiy

**Affiliations:** 1 Department of Modeling of Physical Processes and Systems, University of Tyumen, 6 Volodarskogo Str., 625003 Tyumen, Russia; 2 Tyumen Petroleum Research Center, 79/1 Osipenko Str., 625003 Tyumen, Russia; 3 Rosneft Oil Company, 26/1 Sofiyskaya Naberezhnaya Str., 625000 Moscow, Russia

**Keywords:** gelling agents, incremental oil production, mass conservation law, flow characteristics, conformance control coefficient, injectivity drop

## Abstract

The paper describes the introduction and estimation of performance criteria for the gelling agent injection technology based on a general approach to modeling physical and chemical enhanced oil recovery (EOR) methods. The current mathematical models do not include performance criteria for the process of gelling agent injection and do not allow for assessing the level of success of a treatment job in production wells. The paper introduces such criteria for the first time. To simulate the effect on injection wells, the mass conservation laws and the generalized flow law are used, and closing relations for the gelling rate are taken into account. A conformance control coefficient is introduced which characterizes the positive effect of well treatments and injectivity drop which characterizes the negative effect. The performance criteria allow for identifying the wells where the treatment jobs were the most successful. The model verification, based on the comparison of post-treatment injectivity estimated in the developed model, with Rosneft’s field data showed a satisfactory match. The developed correlations can be used as the basis for a surrogate model that allows for avoiding building sector geological and flow simulation models of the treated zone.

## 1. Introduction

Flooding of oil reservoirs is a conventional technology of secondary oil recovery. It addresses two main issues: maintenance of reservoir pressure (and, therefore, the flow rates of production wells), and the displacement of oil from the rock pores [1]. However, due to vertical and lateral heterogeneities of reservoirs, the behavior of the water–oil displacement front is rather complex. Such complex behavior leads to an early breakthrough of the injected water into production wells. Oil production with high water content shows low performance and incomplete sweep efficiency.

The term “water control” includes defining the field zones with minimal fluid cross-flows, measures to control water injection into various injection wells, and controlling the voidage replacement by injecting water in certain zones [2]. Recently, a new term has been widely used, i.e., “flooding control” or “conformance control”. This term implies comprehensive measures to redistribute flows in a reservoir vertically and laterally and reduce the water cut of recovered fluid resulting from such well interventions [3].

Water-flood management covers two focus areas. The first is stimulating the bottom-hole zone of injection wells and ensuring conformance control between layers with various permeabilities [4]. The response of the nearest producers is determined by a change in lateral piezo-conductivity when forming low-permeable barriers in high-permeable interlayers.

**Figure 1:**
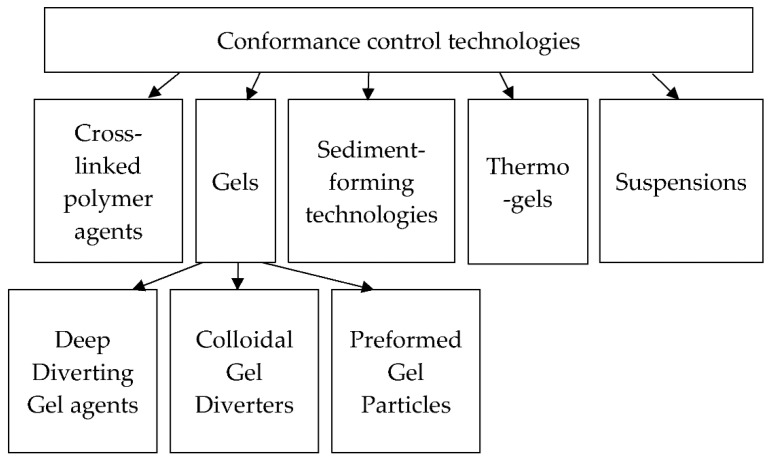
Various conformance control technologies.

However, the presence of vertical cross-flows between interlayers leads to flow forming around these barriers and the return of the flows to their original state. The difference in the rate of vertical conformance and lateral transmission of pressure disturbances leads to a short-term (several months) positive effect on producers (reduction of water-cut, increase in oil rate) [5]. Examples of conformance control technologies or stimulating the zone closest to wells are the injection of cross-linked polymer agents [6,7,8] and sodium-silicate-based gelling agents [9,10,11,12,13], sediment-forming technologies [14], the use of thermo-gels [15,16] and injection of suspensions [17], etc. The examples are shown in Figure 1 below.

The second focus area is related to reservoirs with local highly permeable channels between injection and production wells. Examples of such irregularities are “super-reservoirs” with permeabilities that differ from the average field values by tens or hundreds of times, regional or induced fracturing of formations. Examples include the local occurrence of a super-reservoir at the Talinskoye Field [18] or the development of induced or so-called auto-fractures at the Priobskoye Field, where the water breaks through into fractured producers due to the convergence of two types of fractures [19]. Manipulating these causes of reduced reservoir sweep is called flow-diverting technologies, although the agent bases of conformance control and injected water diverting technologies are very close, as a rule, only the content and injection volumes differ.

The flow-diverting technologies allow the injected agents to be pushed deep enough into the formation or effectively block the fractures without a noticeable impact on the formation matrix. For this purpose, the same suspension agents are used, Deep Diverting Gel agents [6], such as Colloidal Gel Diverters [20], and Preformed Gel Particles [21,22].

The considered approach includes a model of “deep-bed suspension migration” [23] and a general compositional model with chemical reactions [24]. In the first model, the “active” component is suspended particles; in the compositional simulator, the principal components are dissolved substances reacting in porous media. Several analytical solutions were obtained for 1D problems in the framework of deep-bed suspension migration [25].

The 1D objective of oil displacement from a homogeneous reservoir by polymer solution is a “classical” problem [26] and has an analytical solution, which was used to solve an inverse problem for adsorption parameters determination in this paper. For relatively low agent injection volumes in a layered reservoir, vertical fluid cross-flows are negligible in comparison with lateral flows. Therefore, the 1D approximation approach can be applied. This approach allows for considering the conformance improvement problem as a set of 1D objectives in each interlayer without cross-flows [27].

With relatively high treatment performance (more than 1500 m^3^ of incremental oil per well treatment), the success rate for the analyzed sample does not exceed 70%. The reason for this is likely due to the insufficient attention to the geological and physical features of the wells selected for treatment and the non-optimal process parameters of treatment jobs. The solution to this problem is the preliminary designing of treatment jobs based on mathematical models of the process.

Two reasons hinder the use of special reservoir models for treatment design purposes. The first is the high time and financial costs, the second is the fact that the objectives of agent injection to form low-permeable barriers and the response of production wells are of different scales. To solve this problem, the authors suggest applying a combined approach or a “surrogate” model.

This paper discusses only the conformance control technologies. Such an approach allows us to estimate and optimize the technologies to maximize the flow redistribution effect. Another application of such models includes evaluating and comparing the treatment performance under certain geological and physical conditions.

## 2. Results and Discussion

### 2.1. The Influence of Slug Volume

**Figure 2:**
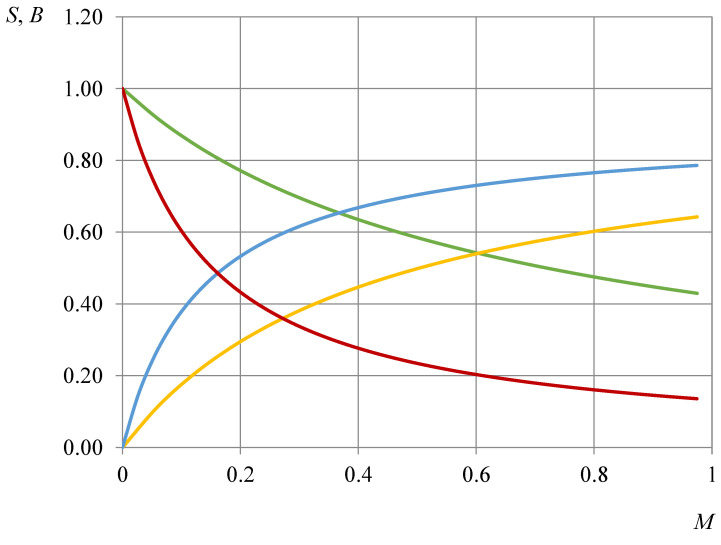
The correlation of CC performance criteria and the dimensionless mass of the injected gelling agent (green line—injectivity drop *S* for linear inflow, red line—injectivity drop *S* for radial inflow, yellow line—CC coefficient *B* for linear inflow, blue line—CC coefficient *B* for radial inflow).

The influence of slug volume on the introduced coefficients was calculated for a particular well described in Section 4.1 and the treatment parameters, which are mentioned in the Materials and Methods section. The results are presented in Figure 2. For comparison, the same calculations were made for a vertical well without a fracture and are given in Figure 2. For the generality of the results, a dimensionless volume of the gel was introduced: *M =* *β V/V_r_*, where *β* is the rock damage coefficient, *V* is the volume of the injected slug and *V_r_* is the volume of the treated reservoir zone.

The analysis of the resulting correlations shows that with the growth of the injected agent mass, the conformance control (CC) coefficient increases, the flows are redistributed more efficiently, and the injectivity profile is improving; however, this leads to a decrease in the overall well injectivity.

### 2.2. Analysis of Field Experience in Gelling Agents Injection

We analyzed the available data on the use of gelling agents based on HPAA and sodium silicate in recent years at Rosneft’s license blocks in West Siberia. The field data analysis allowed us to identify a few features of applying gelling agents. First of all, we concluded that basically such treatments are used in vertical and directional wells both with and without hydraulic fracturing, as well as in horizontal wells without hydraulic fracturing. Next, we will talk only about vertical and directional wells with and without hydraulic fracturing.

As mentioned in Materials and Methods section, the CC technology performance for injection wells is characterized by two parameters: the CC coefficient, which is an “internal” parameter that is not determined in the field, and the injectivity drop coefficient, which is measured after the treatment. The internal parameter characterizes the processes inside the formation, which can not be accurately measured by production logging tools describing the flow rates through perforation intervals, which poorly correlate with flows in layers with various permeabilities. The injectivity drop coefficient is a well-measured parameter (the rate related to drawdown).

**Figure 3:**
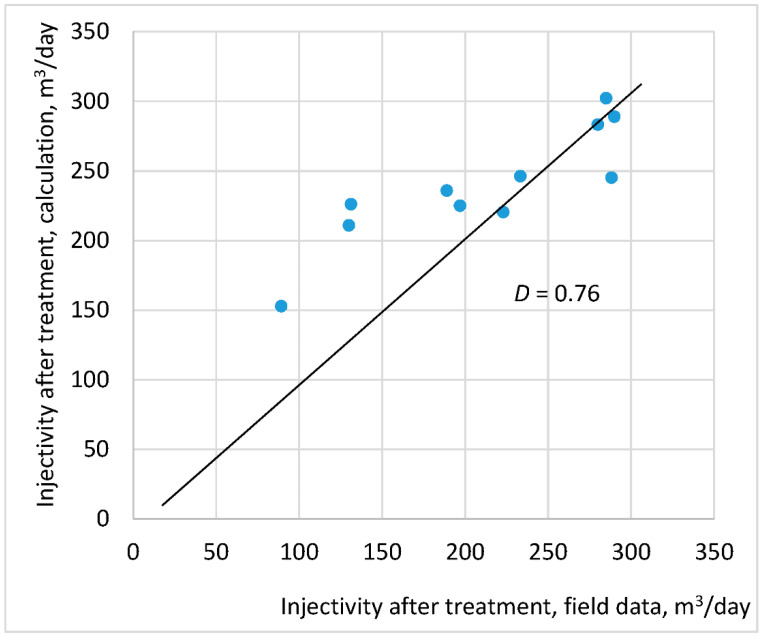
Comparison of post-treatment injectivity, calculated in the developed mathematical model, with its actual value.

Figure 3 shows a cross-plot of the estimates, modeled using the described model, and actual injectivities after the treatment for all well completion types. The cross-plot is described with sufficient accuracy (characterized by the correlation coefficient *D*) by a straight line leaving zero and having an inclination of about 45°, which validates the assumptions of the developed approach. The linear trend is chosen because the field and the calculated data should match. This corresponds to a straight line with an angle of inclination of 45°. The low accuracy of the approximation is associated with the low precision of the field data.

The main point of analyzing the reliability of the ideas embedded in the developed line of gelling mathematical models is to confirm the influence of the formulated criteria on incremental oil production as a result of applying these technologies. For the generality of the results, the processing of dimensionless parameters should be used to characterize incremental oil production from the previously introduced dimensionless criteria for the process’s performance. As such a parameter, it is advisable to use a dimensionless ratio of the incremental volume to the injected volume. We can denote it as *G*.

**Figure 4:**
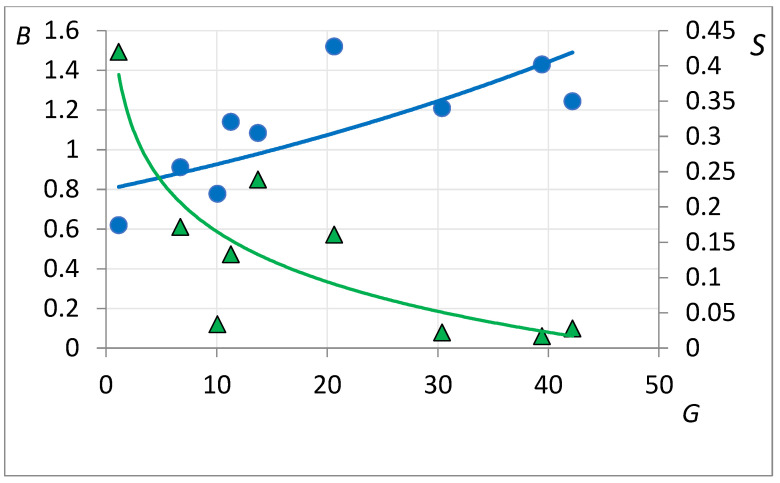
The results of statistical processing of the correlation between the specific incremental production in producers responding to gelling agents injection and the CC coefficient (blue dots and trend) and injectivity drop (green dots and trend).

The developed approach is based on the description of the efficiency of flow redistribution in the interlayers through the CC coefficient. This criterion should have a positive effect on incremental oil production, respectively. To confirm this idea, the incremental production data on vertical and directional injection wells were compared with the estimated CC coefficient (*B*). These calculations are based on well-logging interpretations, as well as the treatment parameters (volumes, compositions, and injection rates). The results of data statistical processing are shown in Figure 4. The resulting function demonstrates a satisfactory correlation coefficient *D* = 0.7.

A negative consequence of the use of gelling agents is a decrease in the injectivity of injection wells, which forces an increase in the BHP to maintain the level of water injection into the reservoir. Statistical processing of data on the effect of the introduced injectivity drop coefficient *S* on dimensionless incremental oil production from applying the gelling technology is also shown in Figure 4. The correlation coefficient is *D* = 0.68. Figure 4 analysis shows that for the considered process, we can set and solve an optimization problem of maximizing incremental oil production with a minimum reduction in well injectivity. Today, the developed approach for the gel and suspension treatment designs is considered in the Rosneft Company. Executed designs are recommended but the field confirmation has not yet been obtained.

## 3. Conclusions

The paper presents a new approach to modeling the conformance improvement processes in the application of gelling technologies in a porous medium with the formation of gel barriers leading to the redistribution of flows in an inhomogeneous reservoir. The approach identifies two related tasks: predicting the processes of forming gel barriers in the bottom-hole zone of a heterogeneous reservoir and the response of production wells to treatment jobs. Analysis of the characteristic times of various effects shows that the first problem can be considered in the framework of a one-dimensional flow in non-connected interlayers. The second task can be considered on the basis of a statistical analysis of the field experience in applying gelling technologies.

In the first problem, the efficiency of flow redistribution in the bottom-hole zone can be characterized by the difference in the root-mean-square deviation of the interlayers’ permeability and the average value before and after the treatment or by the CC coefficient, which was introduced for the first time. It is established that the CC coefficient increases with the increase in the slug volume. The negative effect of the well treatment is manifested through a decrease in the reservoir injectivity. These coefficients describe the integral effect of near-wellbore flow distribution.

The experimental procedures for the model’s parameter determination are considered. The solution of inverse problems was used to find the common time of the gelling reaction, polymer adsorption parameters, and flow characteristics. Examples of such procedures are presented. Unlike the existing approaches, the developed experimental method does not require destructing the core sample to determine the adsorption constants.

The specific effect in the nearest producers from an injector can be characterized by the specific value or the ratio of incremental oil production to the volume of the injected gelling agent. Existing approaches do not contain such characteristics. An analysis of the field experience in applying HPAA/sodium-silicate-based gelling agents showed that the specific incremental oil production will also be determined by the CC coefficient and a decrease in the injectivity coefficient. The developed correlations can be used as the basis for a surrogate model that does not require building geological sector models and flow simulation sector models of the treated zone.

## 4. Materials and Methods

### 4.1. General Approach to Modeling

With all the variety of agents recommended for conformance control (CC) applications, three main groups can be distinguished by the sedimentation/gelling mechanism. The most widely used group includes injection of polymers and cross-linkers. These are partially hydrolyzed polyacrylamides (HPAA) [28,29] cross-linked by polyvalent anions that change valence under reservoir conditions, or organic cross-linkers having a low kinetic constant [24]. For some cases, biopolymers [30] or other types of polymers are recommended.

The second group includes gels formed at high reservoir temperatures [14,15] or preformed gels that swell at elevated temperatures [6,22,31,32]. Sometimes the temperature triggers a primary reaction, the products of which create conditions for gelling [15].

The third group includes agents that form an insoluble precipitate during reaction under reservoir conditions. Most of those agents contain sodium silicate as the main agent reacting with calcium chloride [4]. Somewhat apart in this group is the use of suspended particle systems, the main difference of which is that the particles are captured in a porous medium much faster than chemical actions occur and the particles can break away into the flow due to a change in the liquid flow rate [23].

Thus, the main feature of these processes is the presence of two principal components dissolved in water. For suspensions, the principal component in the mixture is particles that are trapped in a porous medium to form a precipitate. Polymers or gels in suspensions have an auxiliary function of stabilizing the injected system and preventing its gravitational segregation.

The conformance control technology consists of injecting into an injection well a slug of agents that form a gel or sediment in a porous medium and form a low-permeable barrier. The slugs used for such purposes have small volumes of up to 2000 m^3^. Such volumes are several orders of magnitude smaller than the volumes of injection sites and surrounding production wells. Therefore, from the point of view of mathematics, the problem of a production wells response is a small-parameter problem.

It is impossible to find a solution to such a problem in the general formulation, even with a sector flow simulation model. In such cases, the general task is divided into two components. The first is to analyze the distribution of gel or sediment in the bottom-hole zone of injection wells and determine the quantitative characteristics of the redistribution of flows in the bottom-hole zone. The second is the response of production wells taking into account these characteristics.

Before solving these problems, it is advisable to estimate the time of lateral transmission of the pressure disturbance within the reservoir system and the time of gravitational vertical flows. The lateral transmission of pressure disturbances is described by the piezo-conductivity equation, where we can assume that the piezo-conductivity coefficient is determined by the average lateral reservoir permeability $k_{x}$. The viscosity of a reservoir fluid mixture is denoted as *µ_ef_*, and the compressibility of a saturated porous medium is $\beta_{l}$. Then, the common time scale of pressure transfer or flow redistribution is estimated as

(1) $$\tau_{p}\sim \frac{L^{2}\beta_{l}\mu_{ef}}{k_{x\;}},$$

where *L* is the typical size of a problem.

The time of the gravitational flow of fluid between the layers is determined by the Buckley–Leverett equation where the pressure drop is due to the difference in the fluid densities Δρg, where Δ*ρ* is the difference in the densities of water and oil, and *g* is the acceleration of gravity. The flow time can be estimated as

(2) $$\tau_{S}\sim H\emptyset \frac{\mu_{ef}}{k_{y}\Delta \rho g},$$

Here, *Ø* is the average reservoir porosity, *k_y_* is vertical reservoir permeability, and *H* is reservoir thickness; the effective viscosity is determined by the formula:

(3) $$1/\mu_{ef}=\left(f_{w}/\mu_{w}+f_{o}/\mu_{o}\right)\partial F/\partial S,$$

where *f_w_*, *f_o_*, *μ_w_*, *μ_o_* are water and oil relative permeability and water and oil viscosity and *F* is the Buckley–Leverett function.

Let us consider the tasks set for the reservoir with the following parameters: *L* = 1000 m, *H* = 50 m, *Ø* = 0.2, anisotropy $a=\sqrt{k_{y}/k_{x\;}}=0.1,$ *β_l_* = 10^–7^ Pa^–1^, *μ_ef_* = 1 mPa · s, *g*Δ*ρ* = 1000 Pa/m. The time of gravitational cross-flows for such a reservoir will be 1–2 years and the time of the pressure disturbance propagation is 10 times less. Thus, in the first task of injecting agents into the reservoir, it is possible to neglect the cross-flows between the layers, i.e., consider them isolated, and consider the flow radial near vertical or directional wells and linear near hydraulic fractures. In the second task, it is possible to estimate the time during which the effect of the redistribution of flows will be noticeable, which is about a year.

As a result, we can consider the first task in the simplified formulation of single-phase water flow at residual oil saturation as a one-dimensional statement (radial or linear, depending on the well completion type) for all layers of the reservoir separated from well-logging interpretations. Let us first consider this problem for a single interlayer.

As noted above, for the principal gelling components, the continuity equations that determine the kinetics of reactions may be written, as well as the continuity equation for immobile gel or sediment. The generalized Darcy’s law is used as the momentum conservation equation for slow flows. The closing conditions are the laws of the chemical reaction kinetics. Let us consider these equations using an example of two components flowing to form a gel. The mass conservation equations for a multicomponent mixture are written for the mass concentrations of the components in question:

Compositional or multiphase: multicomponent flow models are a common tool for the simulation of fluid flow in reservoirs. These models use the standard specification of specific phase density in terms of saturations and component mass concentrations. Heterogeneous mechanics introduce the concepts of volumetric content of phases and mass concentration of components dissolved in a phase, as well as a pseudo homogeneous mixture in which the velocities of the carrier phase and suspended particles coincide. The latter is often called advective flow. As a result, under the condition of the incompressibility of fluid and rock matrix, the laws of mass conservation are reduced to equations for mass concentration of the components in question:

(4) $$\frac{\partial (\emptyset -\sigma )\left(1-S_{or}\right)c_{i}\rho_{w}+a_{i}\left(1-\emptyset \right)\rho_{r}}{\partial t}+\mathrm{div}\left(\vec{U}c_{i}\rho_{w}\right)=-K_{i}\rho_{w}j,$$

where *c_i_* is the mass concentration of the *i*-th component in the carrier phase (*© =* 1, 2); *a_i_* is the concentration of the adsorbed *i*-th component of the polymer; *σ* is the volume fraction of gel in a single volume of a saturated porous medium; *S_or_* is residual oil saturation of the porous medium; *ρ_w_*, *ρ_r_* are the density of the water phase and porous medium matrix; *U* is the flow rate; *K_i_* is the mass fraction of the *i*-th component necessary for the formation of 1 kg of sediment or gel; *j* is the rate of precipitate formation/gelling; and *t* is time.

The profile of the change in the gel mass per unit volume of the entire saturated porous medium is written as:

(5) $$\frac{\partial \left(\sigma \rho_{g}\right)}{\partial t}=\rho_{g}j,$$

where *ρ_g_* is gel density.

The generalized flow law takes into account the change in flow resistance due to the formed gel:

(6) $$\vec{U}=-\frac{k\;k_{r}\left(S_{or}\right)}{\mu R_{f}\left(\sigma ,a_{i}\;\right)}\mathrm{grad}\;P,$$

where *k* is the absolute permeability; *k_r_*(*S_or_*) is the phase permeability of water at residual oil saturation; *μ* is the viscosity of the injected solution; $R_{f}\left(\sigma ,a_{i}\;\right)$ is the resistance factor in the zone of formation of a low–permeable barrier; and *P* is the pore pressure.

The closing relations for the gelling rate in the approximation of an elementary homogeneous one-sided chemical reaction in a closed system proceeding at constant volume and temperature will be written in the form of Guldberg and Waage’s Law of Mass Action and the reaction constant through the Arrhenius law [33]:

(7) $$j=Z\prod_{i}c_{i}^{n_{i}},\;\;Z=Z_{0}\exp\left(-\frac{E}{RT_{0}}\right),$$

where the $c_{i}^{n_{i}}$ function determines the probability of finding the number of molecules of the *i*-th component necessary for the reaction at a given point in space; *n_i_* is the stoichiometric reaction coefficient (the number of molecules involved in the reaction) for the *i*-th component; *Z* is the reaction constant; *Z*_0_ is the kinetic coefficient; *E* is the reaction activation energy; *RT*_0_ is the energy of molecules thermal motion; *R* is the universal gas constant; and *T*_0_ is the temperature. Note that the complex nature of the reaction can manifest itself in fractional values of *n_i_* and the need to determine the empirical value of the reaction constant. A similar approach to describe the polycondensation reaction is presented, for example, in [21].

The functional relationship of the resistance factor on the sediment or gel in a porous medium is described by different functions. This is due to the fact that there is extremely little special research in this area. Here are just a few examples of the resistance factor as a function of the volume content of precipitate in the rock [5]:

(8) $$R_{f}=\left(1+\beta \sigma \right),\;\;\;\;\;\;\;R_{f}={\left(1+\beta \sigma \right)}^{\gamma },$$

where *γ* is the exponent and *β* is the rock damage coefficient.

The description of suspension flow also fits into the framework of the proposed approach. Suspension consists of micron particles of clay, chalk or wood flour in water, stabilized from gravitational segregation by additives of polymer or weak gel. The migration of particles in a porous medium is described in the framework of a homogeneous multiphase medium in which the velocities of the carrier and dispersed phases coincide. The fraction of particles in the flow is recorded through the mass concentration of the suspension and the particles trapped in the porous medium through the volume fraction in a single volume of the entire porous medium. Such a model is called deep bed suspension migration. The main conditions for its application are a small particle size (smaller than the pore size), and small suspension concentrations (significantly less than the porosity value).

The mechanism of particle capture may vary, but its nature is not chemical, so the trapping kinetics is usually considered proportional to the particle flow modulus:

(9) j=λUc,

where *λ* is the flow coefficient and *c* is the concentration of suspension particles.

This model of suspension flow is widely used to describe the processes of propagation of small particles in a porous medium [23].

When gelling agents are injected into a layered reservoir, they are unevenly distributed over the productive interval: a larger volume of agents enters the highly permeable layers, and a smaller volume, respectively, enters the less permeable ones. Therefore, larger low-permeable barriers will be formed in high-permeable interlayers.

As an example, let us consider the process of HPAA and chrome acetate injection into a vertical well with a fracture with *l* length in layered strata with reservoir net pay *H_ef_*. The reservoir consists of *N* layers with corresponding thicknesses *h_n_*, porosities *Ø_n_* and permeabilities *k_n_*, where *n* identifies the layer’s number.

The governing equations in each layer are as follows:

(10) $$\emptyset_{n}\frac{\partial c_{1n}}{\partial t}+\mathrm{div}\left(U_{n}c_{1n}\right)=-c_{10}c_{20}Zc_{1n}$$

(11) $$\frac{\partial \sigma_{n}}{\partial t}=c_{20}zc_{1n}$$

(12) $$U_{n}=-\frac{k_{n}\;k_{r}\left(S_{or}\right)}{\mu \left(1+\beta \sigma_{n}\right)}\mathrm{grad}P$$

Here, *c*_10_ and *c*_20_ are the initial polymer and chrome acetate concentrations.

The initial conditions for the equation set are the absence of components in a reservoir, the boundary conditions are constant injection rate *Q*_0_ and agents concentrations *c*_10_ and *c*_20_ in the well. The analytical solution for the initial and boundary conditions of the model (10)–(12) is as follows:

(13) $$\sigma_{n}=\frac{Zc_{10}c_{20}V_{inj}}{Q_{0}}\exp\left(\frac{2HlZc_{10}c_{20}\sum_{m}k_{m}h_{m}}{Q_{0}k_{n}h_{n}}x\right),\;\;\;\;\;\;\;\;\;\;\;\;x>0$$

where *V_inj_* is the agent slug volume; *Q*_0_ is the injection rate; *x* is the linear distance from the fracture; and *n* and *m* are the layer’s indicators.

The method and procedure of the governing equations’ solution are presented in the authors’ previous paper [27].

The values of layer permeability after treatment $k_{n}^{*}$ are calculated through the provided analytical solution [27] of gel distribution *σ_©_*). For the linear flow near the fracture, for example, this formula has the view:

(14) $$k_{n}^{*}=\frac{k_{n}r_{c}}{\int_{0}^{L}\left(1+\beta \sigma_{n}\right)dx}$$

where *r_c_* is the mid-length between the injector and the nearest producer.

The methodology for assessing the redistribution of the injectivity profile is given, for example, in [27]. Analytical solutions on the distribution of gel or precipitations in each layer of the reservoir system allow for estimating additional hydraulic resistance. Based on the additional resistance, it is possible to estimate the mathematical expectation of the redistribution of flows near an injection well.

The authors recommend characterizing the reservoir heterogeneity through the mean-square deviation of the interlayers permeability:

(15) $$B_{0}=\sqrt{\frac{1}{N}\underset{n}{\sum }{\left(\frac{k_{n}H_{ef}}{\sum_{m}k_{m}h_{m}}-\frac{k_{a}H_{ef}}{\sum_{m}k_{m}h_{m}}\right)}^{2}};\;B_{1}=\sqrt{\frac{1}{N}\underset{n}{\sum }{\left(\frac{k_{n}^{*}H_{ef}}{\sum_{m}k_{m}^{*}h_{m}}-\frac{k_{a}^{*}H_{ef}}{\sum_{m}k_{m}^{*}h_{m}}\right)}^{2}},\;B=B_{0}-B_{1},$$

where $k_{a},\;k_{a}^{*}$ is the arithmetic mean of permeability before and after the treatment job, *B*_0_ and *B*_1_ are the difference in the mean-square deviations of the interlayers permeabilities before and after the treatment job $k_{n}$ and $k_{n}^{*}$ are permeabilities before and after the treatment job; *h_m_* is the thickness of the *m*-th interlayer; and *N* is the number of interlayers. For brevity, the difference between these indicators *B* is called the CC (conformance control) coefficient. The growth of parameters characterizing the reservoir heterogeneity shows an increase in the CC performance.

The negative effect of treatments is associated with a drop in injectivity of injection wells due to deteriorating reservoir properties after the treatment due to partial blocking of the porous medium channels by gel. Therefore, the second important parameter characterizing the negative effect of treatments should be an injectivity drop:

(16) $$S=\frac{PI_{1}}{PI_{0}},\;PI_{1}=\frac{Q_{1}}{\Delta P_{1}},PI_{o}=\frac{Q_{1}}{\Delta P_{o}},$$

where *PI*_1_ and *PI*_0_ are injectivities after and before the treatment; *Q*_1_ and *Q*_0_ are injection rates before and after the treatment; and ∆*P*_1_ и ∆*P*_0_ are pressure differences between the bottom-hole and the reservoir (at the distance *r_c_*).

As an example of the algorithm for calculating the parameters of flow redistribution in the bottom-hole zone of an injection well, let us consider a treatment job in a vertical well with a fracture of length *l =* 20 m intersecting a reservoir with net pay *H* = 4.7 m, containing seven interlayers with the following parameters: *k_n_* = 5 mD, 100 mD, 15 mD, 50 mD, *h_n_* = 1 m, 0.8 m, 0.7 m, 2.2 m, *k*(*S_or_*) = 0.2. HPAA and chromium acetate solution with initial concentrations $c_{10}=0.1,\;c_{20}=0.091$ were injected as slug *V_o_* = 600 m^3^ into the well, the initial viscosity of the solution was close to the water viscosity of *µ* = 1 cP. The following values were accepted for the HPAA adsorption and reaction constants: *a_imax_ =* 10^–7^, *Z* = 0.002 s^–1^, *β* = 21.

**Figure 5:**
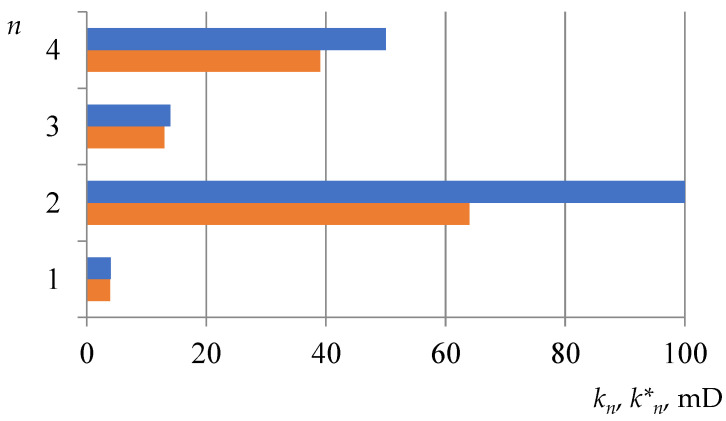
Calculated data on permeability $k_{n}$, $k_{n}^{*}$ change due to gel treatment for concrete well (blue line stands for *k_n_*, orange line stands for $k_{n}^{*}$ ).

Application of the described procedure resulted in a CC coefficient of 0.34 and an injectivity drop factor of 0.74. The redistribution of layers’ permeabilities is shown in Figure 5.

**Figure 6:**
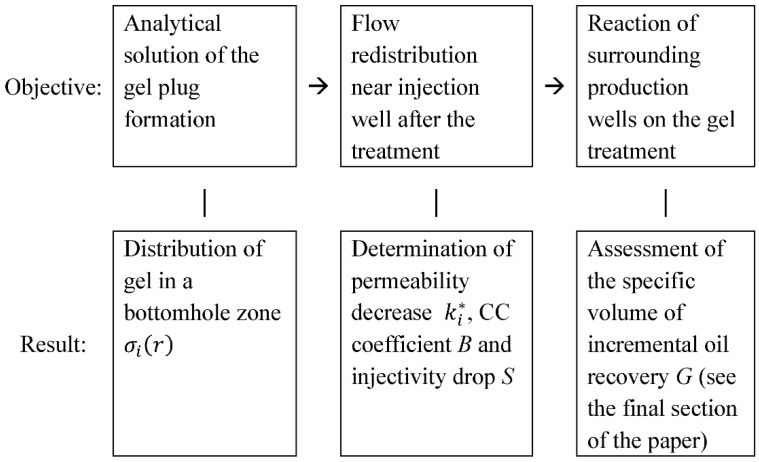
The general workflow to estimate the treatment process parameters.

The schematic view of the key objectives and the whole solution process are demonstrated in Figure 6.

### 4.2. Experimental Determination of Constants Closing the Described Models

Let us consider schematically the gelling reaction of partially hydrolyzed polyacrylamide and, for example, a chromium-acetate-based cross-linker:

(17) $$n_{1}\cdot M_{p}+n_{2}\cdot M_{Cr}+n_{3}\cdot M_{w}\to n_{4}\cdot M_{g},$$

where *M_p_*, *M_Cr_*, *M_w_* and *M_g_* are the averaged analogs of the masses of polymer, chromium acetate, water, and gel molecules. It is assumed here that the polymerization reaction or the formation of the gel mesh structure occurs when interacting with polyvalent metal anions with water absorption into the mesh structure. The mass ratio of the reacting components cannot be calculated or determined from this equation. However, a classical theory of homogeneous reactions allows us to model the process under certain assumptions that simplify further reasoning.

In physical chemistry, the gel point is defined as the time of the beginning of rheological changes in the system. This time is associated with the transition from the formation of linear or branched oligomers or sol to polycondensation of oligomers into two or three-dimensional meshes [34].

For the analysis of the gelling process kinetics, a more important time is the characteristic gelling time, which determines the completeness of the gelling reaction [35]. The characteristic gelling time is determined under laboratory conditions and its value is usually estimated by the following: (a) by the ability to hold a stick in an upright position in a test tube with the initial agents, (b) by forming a 15% immobile gel layer on the walls of the glass when pouring the mixture out of it [24], (c) by forming a 5 cm gel column when dipping and lifting the chemical spatula at the mixture level [36]. All methods allow only for estimating the characteristic time. We will conditionally call these methods “bottle tests”.

We can consider the continuity Equations (11) and (12) in the conditions of chemical experiments (in a beaker, test tube, etc.). There is no movement of components in the vessel and the porosity is equal to unity. Then, the defining system of equations using the gelling reaction as an example will take the form:

(18) $$\frac{dc_{i}}{dt}=-K_{i}j,\;\;\;\;\;\;\frac{d\sigma }{dt}=j,\;\;\;\;\;\;j=Zc_{1}^{n_{1}}c_{2}^{n_{2}}c_{3}^{n_{3}},$$

where it is assumed that the water phase density is close to the gel density *ρ_g_* ≈ *ρ_w_*.

An example of a partially hydrolyzed polyacrylamide (PAA) cross-linker, as mentioned earlier, is chromium acetate, and a change in its valence under reservoir conditions is the triggering mechanism of the reaction. Sometimes organic cross-linkers are used to form gels that are more resistant to reservoir conditions [24].

Let us estimate the mass fractions of the components in the Equation (13) reaction. The average molecular weight of polymer molecules is thousands of grams of molecules and the proportion of water in gelling process is high (more than 90% of the gel consists of water), therefore, the mass fractions of these agents in the reaction significantly exceed the proportion of the cross-linker.

Thus, we can assume that the reaction proceeds with an excess of water (*c*_3_ ≈ 1, *c*_1_ ≈ 0.001, and *c*_2_ ≈ 0.001), and the consumption of the cross-linker is insignificant (*K*_2_ → 0, *c*_2_ ≈ *c*_20_). We will also consider the reaction to be of the first order for the polymer. Thus, the rate of the chemical reaction will be approximated by the expression: *j* = *Zc*_1_*c*_20_. Analysis of the mass balance of the chemical reaction shows that this approach can also be applied to sodium-silicate-based gelling systems [37]. These approximations make it possible to obtain analytical solutions for estimating the kinetic constant.

Let us consider the bottle test results for the studied system, described in [24]. The initial agents were as follows: partially hydrolyzed PAA (25% degree of hydrolysis) with a molecular weight of 20 molecular-mass distribution (MMD), with a mass concentration of 0.003, polyethyleneimine was used as an organic cross-linker that allows for increasing the temperature of gel destruction, with a concentration of 0.0005 in water. In the above study, the results obtained by various methods were compared and the most accurate method was identified with the outflow of reaction products from a chemical beaker and forming of 15% gel on the beaker walls.

The solution of the system of Equation (4) with a simplified setting of the chemical reaction rate is transformed to:

(19) $$c_{1}=c_{10\;}\exp\left(-\left(K_{1}Zc_{20}\right)t\right),\;\;\;\;\;\;\sigma =\frac{c_{10}}{K_{1}}\left(1-\exp\left(-\left(K_{1}Zc_{20}\right)t\right)\right).\;$$

Here, the initial conditions are used when the polymer concentration is equal to *c*_1_ = *c*_10_ and the gel concentration is *σ* = 0. According to the solution, for a sufficiently long time, the volume fraction of the gel is equal to unity, provided the reaction is complete. In this case, the condition for the reaction completeness is equality: *K*_1_ = *c*_10_. Usually, the initial composition of the solution is selected from the condition of reaction completeness, so further we will assume that the polymer mass fraction in the reaction is determined by its initial concentration.

The solutions of Equation (15) are called kinetic curves. With a volume fraction of the gel equal to 0.15, the characteristic reaction time *τ* or the reaction constant *Z* = (*c*_10_ *c*_20_ *τ*)^–1^ is determined. Using the data from [24], the following values were obtained: *τ* = 52 h and *Z* = 0.579 s^–1^. In the absence of kinetic experimental data, it is possible to obtain the considered constants from the history matching data [38].

The flow characteristics of the gelling agent are also considered by the example of determining the flow and adsorption characteristics of a polymer–hydrolyzed polyacrylamide (HPAA) [39]. These characteristics are determined from core flow experiments with polymer solution with measuring differential pressure drop and, if possible, measuring the polymer concentration in the outgoing flow.

After pumping a sufficient volume of polymer solution (pressure stabilization), the resistance factor *R*(*a*) is determined, where *a* is the concentration of the adsorbed polymer. The transient flow regime is compared with the classical solutions for water displacement by the polymer solution, which allows for determining the adsorption characteristics of a porous medium. Analysis of the flow of the tail and leading edges of the polymer slug also allows us to conclude that the pore volume is inaccessible to the polymer [36].

**Figure 7:**
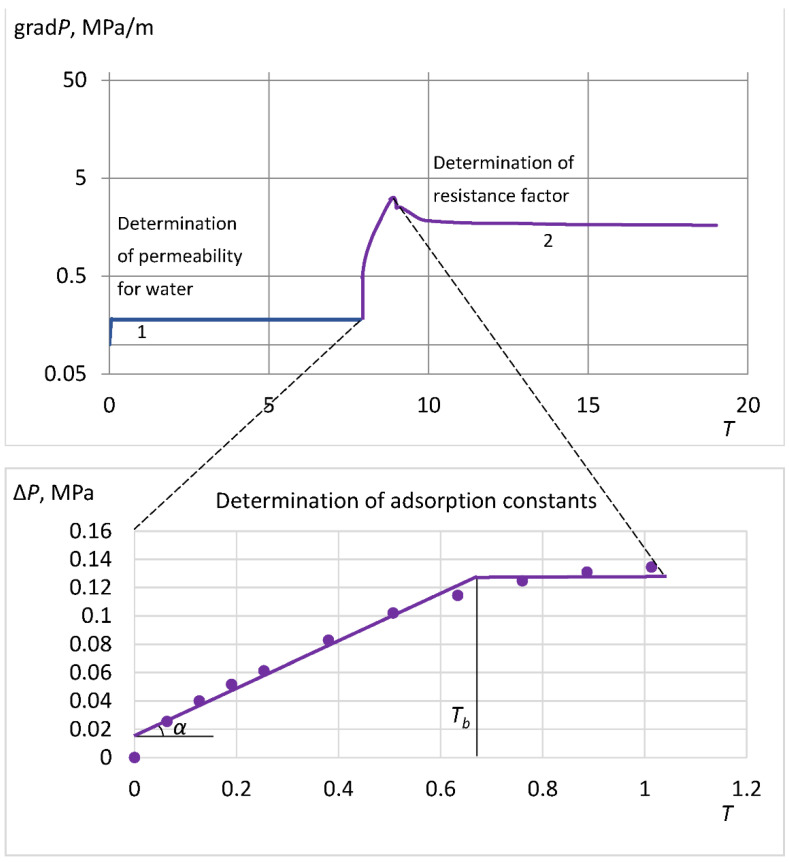
Graphical solution of inverse problems to determine the polymer adsorption parameters (stage 1 and blue line—determination of water permeability, stage 2 and purple line—determination of the resistance factor).

An example of solving these inverse problems is schematically shown in Figure 7, where Δ*P* is the pressure drop, *T* is the number of pumped pore volumes, *T_b_* is the breakpoint, and *α* is the inclination angle. The formulation of inverse problems and their solution are presented in [26,39].

The paper [38] describes a similar approach to determine the flow parameters of the suspension *λ* and *β*. In the absence of experimental data, characteristics can be obtained from solving a more complex inverse problem of treating the bottom-hole zone of an injection well for a reservoir consisting of non-connected interlayers with various flow parameters [40].

## Data Availability

Not applicable.
